# MLK3 Phophorylates AMPK Independently of LKB1

**DOI:** 10.1371/journal.pone.0123927

**Published:** 2015-04-13

**Authors:** Lingyu Luo, Shanshan Jiang, Deqiang Huang, Nonghua Lu, Zhijun Luo

**Affiliations:** 1 Research Institute of Digestive Diseases, The First Affiliated Hospital of Nanchang University, Nanchang, Jiangxi, 330006, China; 2 Graduate Program, Jiangxi Medical College, Nanchang University, Nanchang, Jiangxi, 330006, China; 3 Institute of Basic Medical Sciences, Nanchang University, Nanchang, Jiangxi, 330006, China; 4 Department of Biochemistry, Boston University School of Medicine, Boston, Massachusetts, 02118, United States of America; Boston University School of Medicine, UNITED STATES

## Abstract

Emerging evidence has shown that cellular energy metabolism is regulated by the AMPK and MLK3-JNK signaling pathways, but the functional link between them remains to be determined. The present study aimed to explore the crosstalk between MLK3 and AMPK. We found that both JNK and AMPK were phosphorylated at their activation sites by TNF-α, Anisomycin, H_2_O_2_ and sorbitol. Interestingly, sorbitol stimulated phosphorylation of AMPK at T172 in LKB1-deficient cells. Following the screening of more than 100 kinases, we identified that MLK3 induced phosphorylation of AMPK at T172. Our *in vitro* analysis further revealed that MLK3-mediated phosphorylation of AMPK at T172 was independent of AMP, but addition of AMP caused a mobility shift of AMPK, an indication of autophosphorylation, suggesting that AMP binding and phosphorylation of T172 leads to maximal activation of AMPK. GST-pull down assays showed a direct interaction between AMPKα1 subunit and MLK3. Altogether, our results indicate that MLK3 serves as a common upstream kinase of AMPK and JNK and functions as a direct upstream kinase for AMPK independent of LKB1.

## Introduction

There are several mitogen-activated-protein kinases (MAPKs) pathways consisting of three tiers of kinases, MAPK, MAPK kinase (MAP2K), and MAPK kinase (MAP3K). When triggered by extracellular and intracellular signals, each is consecutively phosphorylated and activated by its upstream component, leading to an amplification of signaling cascade [1]. MAPK cascades are involved in diverse cellular activities, including mitosis, programmed cell death, motility and metabolism [2]. Substrates for MAPKs include transcription factors, phospholipases, protein kinases, cytoskeleton-associated proteins and membrane receptors.

The mixed-lineage kinases (MLKs) are a family of serine/threonine protein kinases, and the catalytic domain of MLK3 resembles both serine and threonine kinase and tyrosine kinase [3]. MLK belongs to MAP3K that contains four isoforms, MLK1, 2, 3 and 4, all of which encompass an amino-terminal SRC-Homology-3 (SH3) domain, a kinase domain, a leucine-zipper region and a Cdc42/Rac-interactive binding (CRIB) motif [4]. Dimerization is a common mechanism for the activation of MLKs [5]. SH3 domain binds to a proline residue in a region between the leucine zipper and the CRIB motif, which leads to autoinhibition of kinase activity. Disrupting the binging between SH3 and proline residue, for example, deletion of the active leucine zipper [5], binging of GTP-bound Rac or Cdc42 with CRIB [4], results in activation of MLK.

**Fig 1:**
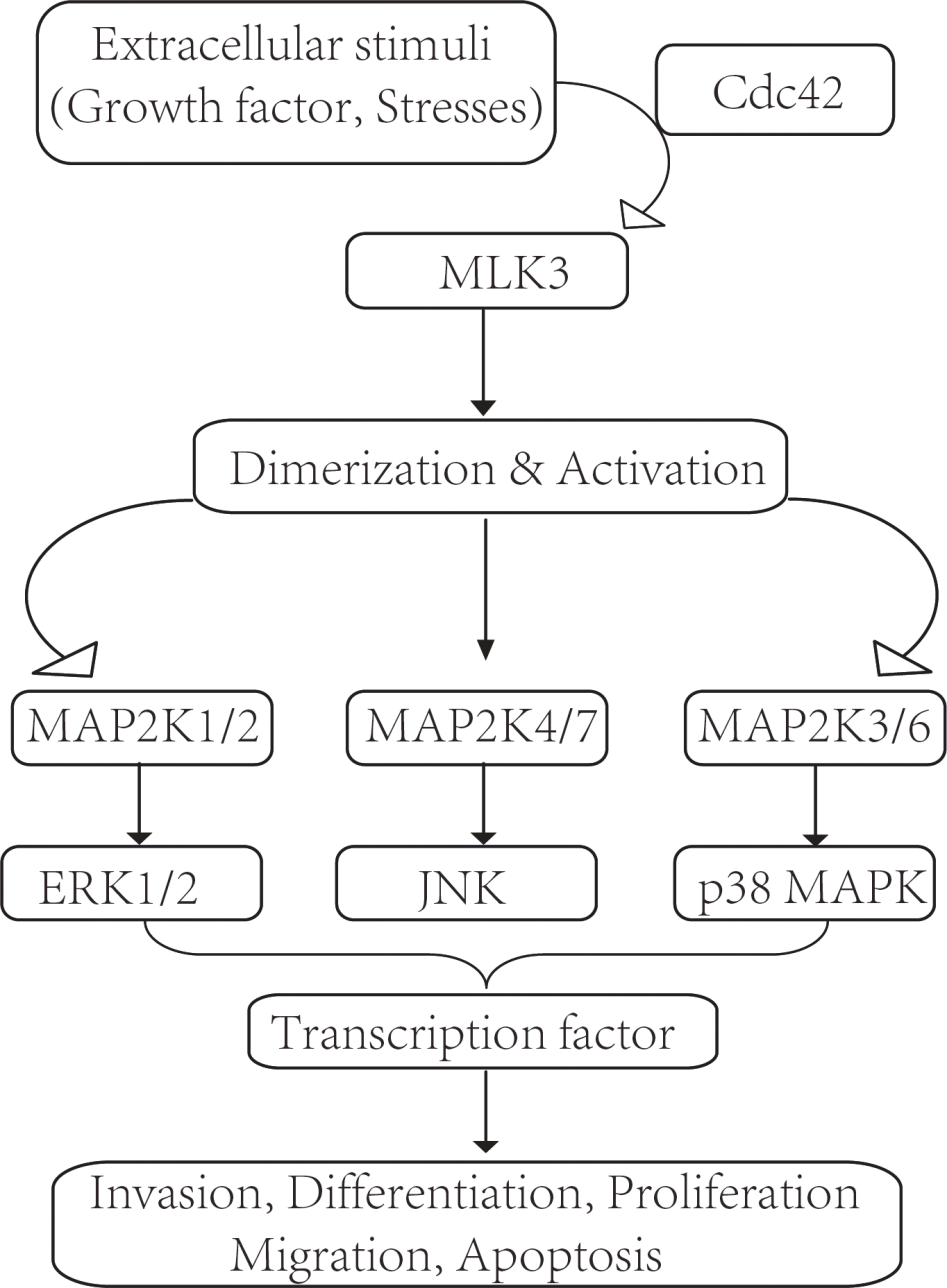
The MLK3 signaling pathway. In response to extracelluar stimuli, MLK3 is dimerized and activated by Cdc42/Rac [5, 8]. Once activated, MLK3 will activate multipe MAP2Ks, which in turn activate the downstream MAPKs by phosphorylation, such as ERK1/2, JNK, or p38 MAPK. The activated MAPKs will further participate in regulating various cellular processes via transcription factors.

Among MLKs, MLK3 is well studied and plays a crucial role in stress and inflammatory responses through regulation of the JNK pathway by phosphorylating MAP2K4/7 and the p38 MAPK pathway by phosphorylating MAP2K3/6 (Fig 1.) [6]. Depending on the cellular context, MLK3 activation can elicit different cellular responses, even opposing biological responses. In neuronal cells, nerve growth factor (NGF) withdrawal activates MLK3-JNK, which in turn mediates neuronal cell death and pathological neurodegenerative diseases, including Parkinson’s disease (PD). Emerging data suggest that blocking MLKs-JNK signaling can prevent the cell death of neurons. For example, MLKs inhibitor, CEP1347, is now in phase II/III clinical trials for neuroprotection in PD [7]. Moreover, MLK3 promotes the cytokine-induced pancreatic beta cell death in type 1 diabetes [8, 9]. In addition, MLK protein could regulate cell cycle and cell proliferation. Finally, MLKs are expressed in cells of the immune system and involved in toll-like receptor mediated signaling pathway.

AMPK is a heterotrimeric kinase composed of α catalytic subunit and regulatory β and γ subunits [10], and plays a key role in the regulation of energy homoeostasis [11]. The activation of AMPK is controlled by the allosteric regulators AMP, ADP and ATP, and phosphorylation of T172 by upstream kinases. AMP binds the γ regulatory subunit causing conformational change in holoenzyme while ATP maintains an inactive conformation. Binding of AMP enables phosphorylation by upstream activating kinases leading to maximal activation of AMPK and impedes dephosphorylation by phosphatases [12]. The upstream kinases thus far reported include LKB1, CaMKKβ, and TAK1 [10].

Cellular functions that are mediated by MLK3, such as inflammation, apoptosis, growth, and differentiation are associated with energy metabolism [5]. Recent studies have revealed that MLK3-JNK pathway plays an important role in the mitochondrial dysfunction and apoptosis [13]. Moreover, AMPK, as a sensor of cellular energy status, is associated with activation of JNK as well as apoptosis [14]. These facts suggest a potential functional link between AMPK and MLK3-JNK in the regulation of cellular energy metabolism.

In the present study, we compared the upstream activators for JNK and AMPK and found that they could be activated by similar factors such as Osmotic stress and oxidative stress. Further mechanistic investigation demonstrated that MLK3 activated AMPK in addition to JNK.

## Materials and Methods

### Materials

Antibodies against p-AMPKα (T172) and pSAPK/JNK were purchased from Cell Signaling Technology (Danvers, MA, USA); Antibodies against AMPKα1, AMPKα2, and LKB1, and recombinant LKB1 protein were purchased from EMD Millipore (Gibbstown, NJ, USA); Antibodies against MLK3 and GST, HRP-conjugated second antibodies and protein A/G agarose were purchased from Santa Cruz Biotechnology (Santa Cruz, CA, USA). Myc antibody was from Sigma-Aldrich (St Louise, MO, USA). 5-amino-1-β-D-ribofuranosyl-imidazole-4-carboxamide (AICAR), Anisomycin, sorbitol, oligomycin, TNFα, IL6 and Adiponectin, glutathione agarose and glutathione were purchased from Sigma-Aldrich (St Louise, MO, USA).

### Cell culture, plasmid transfection and establishment of stable cell lines

The lung adenocarcinoma A549 and human embryonic kidney HEK293T cells were purchased from ATCC (Manassas, VA, USA), and also used in other studies [15]. Cells were cultured in DMEM (Gibco, Life Technologies) supplemented with 10% fetal bovine serum in a 5% CO_2_ and 37°C incubator. The lentivirus carrying LKB1 was packaged in HEK293T cells and supernatant was collected after two days. A549 cells were infected with the supernatant and selected with puromycin [16].

### Western blot analysis

Cells were harvested and lysed in lysis buffer (50 mM Tris-HCl [pH 8.0], 50 mM β-glycerophosphate, 1 mM EDTA, 1 mM EGTA, 1 mM NaVO_4_, 50 mM NaCl, 0.5% Triton x-100, 10 mM NaF, 1 mM DTT, 2 μg/m1 aportinnin, 2 μg/ml leupeptin, 2 μg/ml pepstatin, mM phenylmethylsulfonyl fluoride [PMSF]) on ice for 30 min. Proteins were boiled in 1×SDS loading buffer, separated on SDS-PAGE and transferred onto PVDF membranes (Millipore, Gibbstown, NJ, USA). After blocking in 5% milk for 1 hour, membranes were incubated with primary antibodies at 4 ^o^C overnight. The next day, by incubation with anti-mouse or rabbit IgG secondary antibody, signal was developed with a SuperSignal West Pico Chemiluminescance kit (Thermo-Fisher scientific, Waltham, MA, USA) and exposed to X-ray film.

### Glutathione purification

cDNAs GST-AMPKα1 and GST-MLK3 were cloned in pEBG plasmid and transfected into HEK293T cells. Two days after transfection, recombinant proteins were purified with glutatione (GSH) agarose and eluted with reduced GSH (25 mM).

### *In vitro* phosphorylation kinase assay

Recombinant AMPK was mixed with MLK3 or LKB1 in the presence or absence 5 mM AMP, in a buffer containing 50 mM HEPES, 5 mM MgCl_2_, 1 mM DTT. The kinase assay was initiated by addition of ATP (final concentration of 200 μM) and incubated at 30°C for 30 min. Phosphorylation of AMPK at T172 was determined by western blot analysis.

## Results

### AMPKα can be phosphorylated by sorbitol independently of LKB1

**Fig 2:**
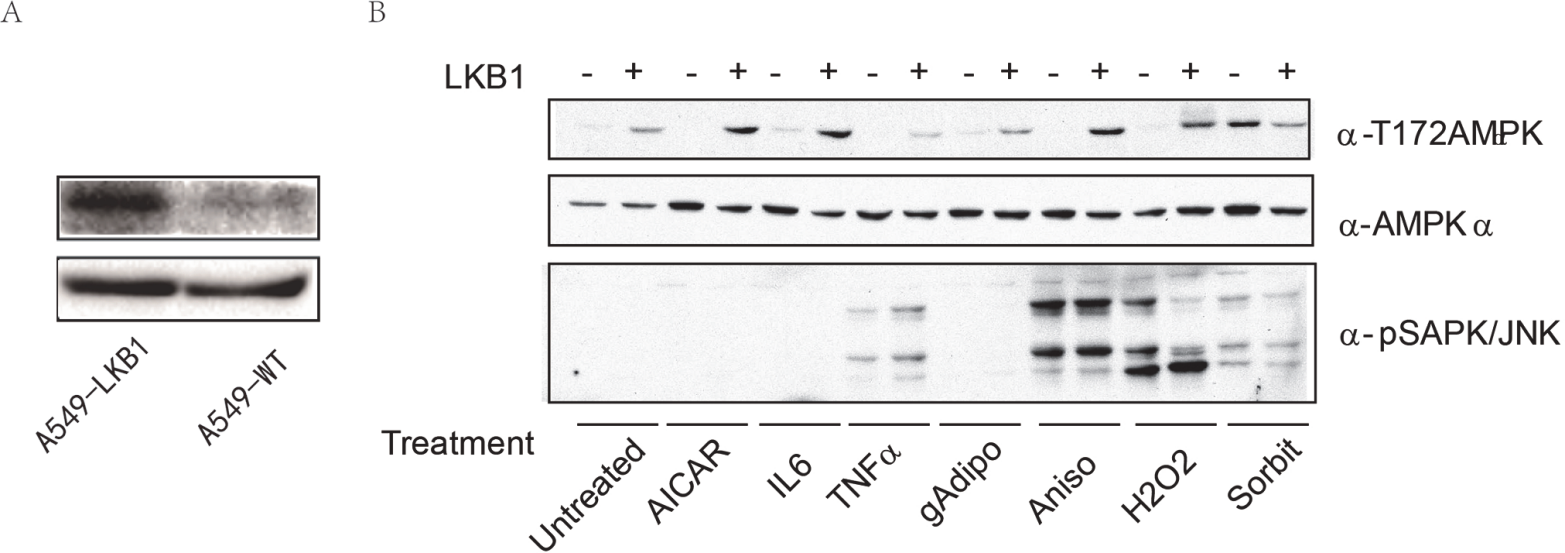
Activation of AMPK and JNK/MAPK by stresses and ligands. A: A stable A549 cell line was established by infection of lentivirus carrying LKB1 cDNA. LKB1 expression was examined by Western blot. B: A549 cells and stable A549-LKB1 cells were treated with IL6 (10 ng/ml), TNF-α (10 ng/ml), Adiponectin (10 μg/ml), Anisomycin (10 μg/ml), H_2_O_2_ (10 mM) and sorbitol (2.5 mM) for 30 min, or AICAR (1mM) for 2 h. Total proteins were extracted and blotted with antibodies as indicated.

To find the potential association of AMPK with MLK3-JNK and to search for additional kinases upstream of AMPK independent of LKB1, we chose lung adenocarcinoma A549 cell line lacking LKB1 and established a stable A549 cell line by infection of lentivirus carrying LKB1 cDNA (Fig 2A). The A549 cells (A549-WT) and A549 cells expressing LKB1 (A549-LKB1) were treated with AICAR, IL6, TNF-α, Adiponectin, Anisomycin, H_2_O_2_, as well as sorbitol. The phosphorylation of AMPKα and JNK was examined by immunobloting. As shown in Fig 2B, TNF-α, Anisomycin, H_2_O_2_, and sorbitol treatment resulted in phosphorylation of SAPK/JNK. All agents stimulated the phosphorylation of AMPKα at T172 in A549-LKB1 cells. Interestingly, sorbitol, which causes osmotic shock, also stimulated the phosphorylation of AMPKα at T172 in A549 cells lacking LKB1 as potently as the cells with LKB1, indicating existence of an additional stress kinase that could phosphorylate and activate AMPK independently on LKB1.

### MLK3 phosphorylates AMPK in the absence of AMP

**Fig 3:**
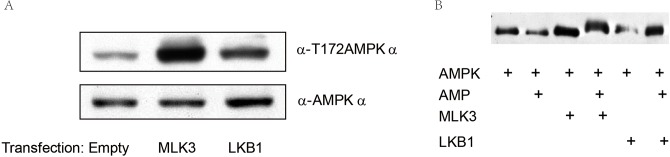
MLK3 phophorylated AMPKα at T172. A: HEK293 cells were transfected with empty control, MLK3 or LKB1 vectors. Total proteins were extracted for immunoblotting of pAMPKα-T172 and AMPKα. B: Recombinant AMPK and MLK3 were respectively expressed in HEK293T cells and purified by GSH. *In vitro* kinase assays were performed as indicated in the presence or absence of AMP. The phosphorylation of AMPK at T172 was determined by western blot analysis.

To search additional kinases that might phosphorylate AMPKα independently of LKB1, we transfected HEK293T cells individually with plasmids of LKB1, TAK1, IKKβ, MLK3, and additional 100 kinases, and determined pAMPKα-T172 status by immunoblotting. Our data showed that besides LKB1, ectopic overexpression of MLK3 led to potent phosphorylation of AMPKα, which was even greater than LKB1 (Fig 3A), indicating that AMPKα can be phosphorylated by MLK3. Interestingly, we could not observe T172 phosphorylation by TAK1 (*data not shown*).

We next performed an *in vitro* kinase assay in which purified kinases were added together in the presence or absence of AMP. To do this, we first expressed MLK3 and AMPK as GST fusion proteins separately, purified the recombinant proteins with GHS agarose beads and eluted them with reduced GSH. GST-LKB1 was used as a positive control. As shown in Fig 3B, phosphorylation of AMPKα1 by LKB1 was increased by addition of AMP. In contrast, MLK3 potently phosphorylated pAMPK-T172 without AMP, but addition of AMP caused an apparently shift in mobility although it did not change the level of pAMPK-T172. These results demonstrated that MLK3 phosphorylate AMPK in the absence of AMP but maximal activation could be achieved by binding of AMP, as suggested by autophosphorylation (a mobility shift).

### MLK3 physically interacts with AMPKα1

**Fig 4:**
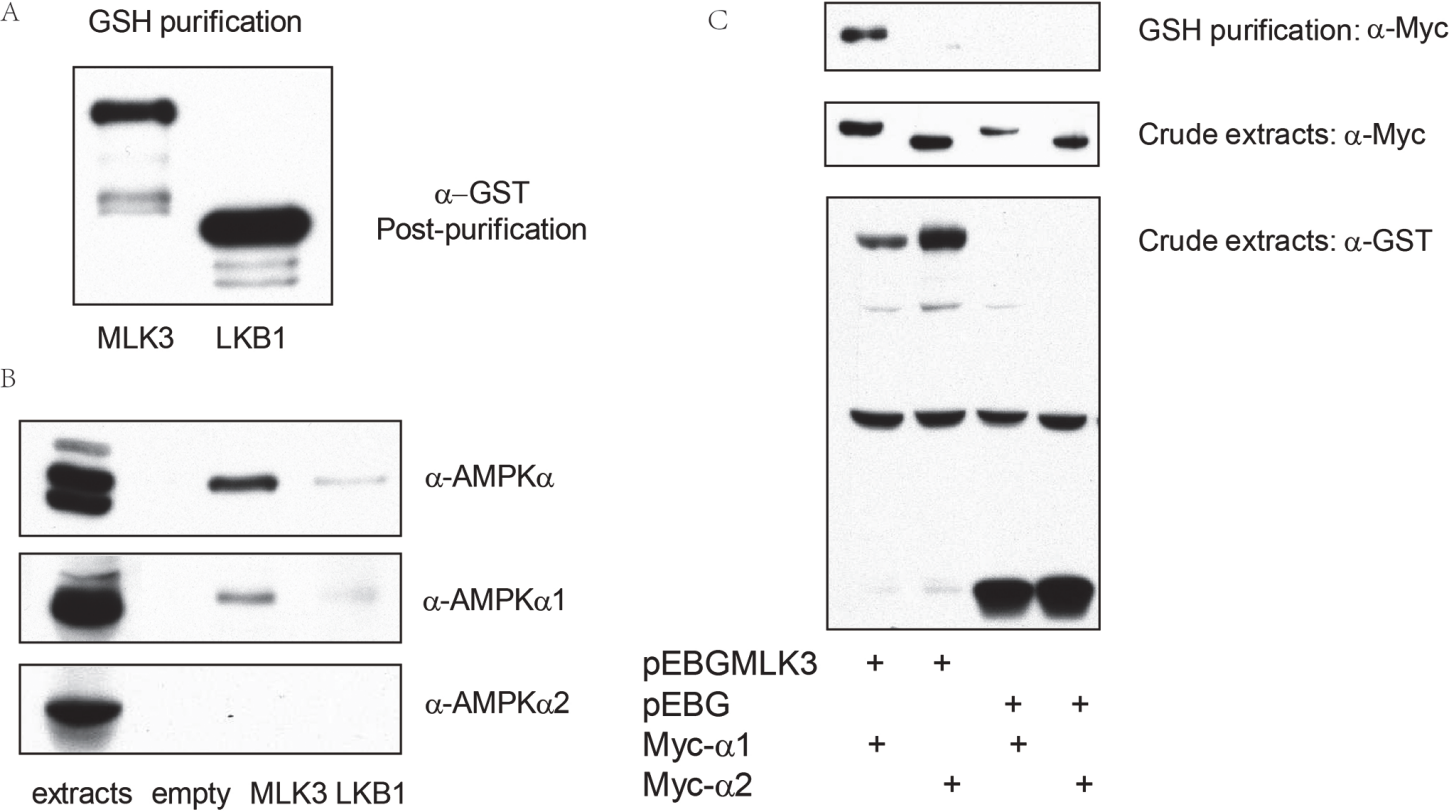
MLK3 physically interacted with AMPKα1. A: HEK293T cells were transfected with GST-MLK3 or GST-LKB1, and then MLK3 and LKB1 were pulled down with GSH beads and blotted with anti-GST antibody. B: HEK293T cells were transfected with GST empty vector, GST-MLK3 or GST-LKB1, and then recombinant proteins were pulled down with GSH beads and blotted with anti-AMPKα, anti-AMPKα1, as well as anti-AMPKα2. C: GST-MLK3 or GST was co-expressed either with Myc-AMPKα1 or Myc-AMPKα2 in HEK293T cells. Immunoblotting was performed on GSH-pulldown and crude extracts with antibodies against GST and Myc, respectively.

To assess if MLK3 directly associates with AMPK, we transfected HEK293 cells with GST-MLK3 or GST-LKB1 (in pEBG), pulled down MLK3 or LKB1 protein with GSH beads, and examined the association of GST-MLK3 or LKB1 with endogenous AMPKα1 or AMPKα2. MLK3 appeared to bind to α1 subunit strongly, as compared with LKB1 (Fig 4A). To further validate the binding of MLK3 to α1, we co-transfected pEBG-MLK3 with Myc-AMPKα1 or α2 in HEK293 cells, purified MLK3 by GSH beads, and then blotted with antibody against Myc epitope. As shown in Fig 4B, MLK3 preferentially associated with Myc-AMPKα1, but not Myc-AMPKα2. This association was not due to Myc-AMPKα1 binding to GST.

## Discussion

**Fig 5:**
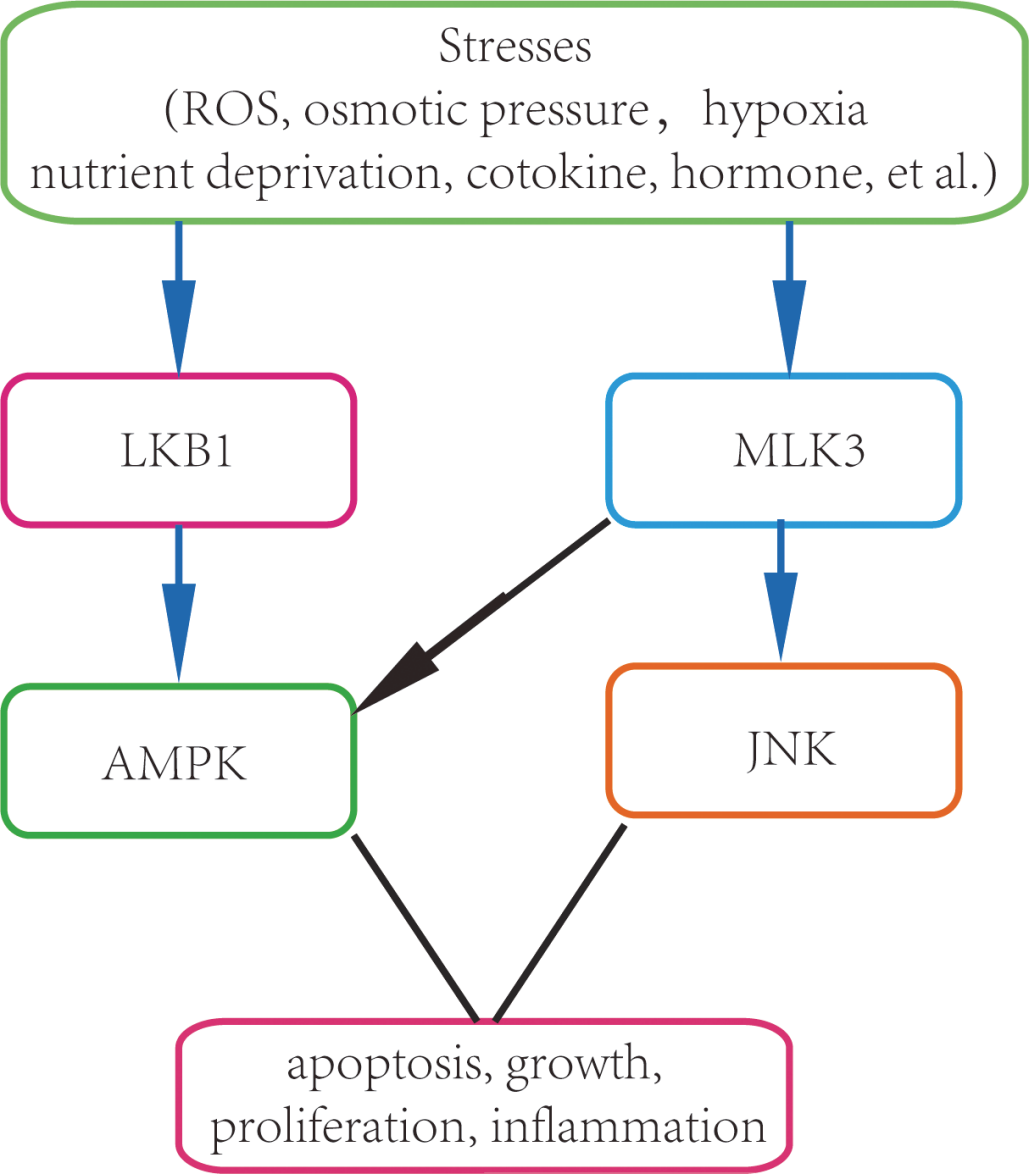
Model of crosstalk between MLK3 and AMPK in stress responses.

Under different stresses, we found JNK and AMPK were often phosphorylated by similar factors. TNF-α, Anisomycin, H_2_O_2_ and sorbitol were able to activate both AMPK and JNK (Fig 2), suggesting there might be a crosstalk between AMPK and MLK3-JNK pathways. Particularly, sorbitol was able to activate the phosphorylation of AMPK in LKB1-deficient cells, demonstrating that sorbitol activates AMPK in an LKB1-independent way. To explore additional stress kinases that might act upstream of AMPK by directly phosphorylating AMPK at T172, we screened more than 100 kinases and identified two kinases, MLK3 and IKKβ (data not shown), which could induce AMPK phosphorylation. Our *in vitro* and *in vivo* analyses further revealed that expression of MLK3 induced phosphorylation of AMPK at T172 in the absence of AMP (Fig 3). Our GST-pull down assays showed a direct interaction between AMPKα1 subunit and MLK3. These results indicated that MLK3 served as a common upstream kinase of AMPK and JNK, and functioned as a direct upstream kinase for AMPK independent of LKB1 (Fig 5.). Comparing the two upstream kinase of AMPK, we found there was indeed some difference between MLK3 and LKB1 in activating AMPK. It is well known that binding of AMP enables phosphorylaiton of AMPK at T172 by LKB1. However, we found that MLK3 phosphorylated AMPK without AMP, but the latter seemed to cooperate with phosphorylation, leading to maximal activation of AMPK. Metformin, a classical AMPK activator, inhibits Complex I of the respiratory chain, and hence activates AMPK by increase the ratio of AMP to ATP; 2-Deoxyglucose is well known for its function in activating AMPK in part by depleting ATP due to its rapid and uncontrolled phosphorylation by hexokinase [10]; AICAR activates AMPK through intracellular conversion to ZMP, an analog of AMP [17]; IL6 can activate AMPK by increasing the concentrations of AMP [17, 18]. Activation of AMPK by the above four drugs depends on LKB1. In agreement, our results demonstrated that the activation of AMPK by LKB1 requires AMP. In all stimuli tested in this study, we only found that sorbitol activated AMPK in the absence of LKB1; whether sorbitol activates AMPK via MLK3 remains to be determined.

In summary, we found that sorbitol activated JNK as well as AMPK independently of LKB1. Most importantly, we discovered that MLK3 phosphorylated AMPK both *in vitro* and *in vivo* and physically associated with AMPKα1. Our study suggests that MLK3 is an upstream kinase that directly phosphorylates AMPK. The AMPK and JNK signaling pathways are both associated with cell proliferation and tumorigenesis. The discovery of MLK3 as a common upstream kinase of AMPK and JNK suggest that activation of MLK3 may not only activate AMPK to inhibit cell metabolism via mTORC1 signaling but also stimulate JNK signal to promote cell apoptosis [19]. Our findings suggest that MLK3 may serve as an anti-cancer drug target.
